# Effect of tegileridine pretreatment on fentanyl-induced cough during general anesthesia induction: a randomized controlled trial

**DOI:** 10.3389/fmed.2026.1866291

**Published:** 2026-07-15

**Authors:** Shaojie Zhang, Jun Hu, Yajuan Zhao, Hongyi Xiao, Yonghao Hou, Huiwen Zheng

**Affiliations:** 1 Department of Anesthesiology, Weifang People's Hospital, Weifang, China; 2 Department of Surgery, Johns Hopkins University School of Medicine, Baltimore, MD, United States; 3 Department of Obstetrics, Weifang Maternal and Child Health Hospital, Weifang, China

**Keywords:** biased μ-opioid receptor agonist, fentanyl-induced cough, general anesthesia, randomized controlled trial, tegileridine

## Abstract

**Background:**

Fentanyl-induced cough (FIC) is a common adverse reaction during anesthetic induction. Although usually transient, FIC may increase intracranial, intraocular, and intra-abdominal pressures and cause serious complications. Tegileridine (SHR8554), a G-protein-biased μ-opioid receptor agonist, may modulate opioid-induced cough, although the underlying mechanism remains unproven. This study evaluated whether tegileridine pretreatment reduces FIC.

**Methods:**

In this prospective, randomized, double-blind, placebo-controlled trial, 232 adults scheduled for elective surgery under general anesthesia were screened, and 216 were randomized to receive tegileridine 0.5 mg diluted to 5 mL (Group T) or 5 mL normal saline (Group C). The study drug was injected intravenously over 10 s immediately before rapid fentanyl injection (4 μg·kg^−1^ over 2 s). The primary endpoint was FIC incidence within 2 min after fentanyl injection. Secondary endpoints included cough severity, hemodynamic changes, and adverse events.

**Results:**

FIC occurred in 31 of 108 patients in Group C and 12 of 108 patients in Group T (28.7% vs. 11.1%; χ^2^ = 10.48, *P* = 0.001). Tegileridine reduced the relative risk of FIC by 61.3% (RR, 0.39; 95% CI, 0.21–0.71), with an absolute risk reduction of 17.6% and a number needed to treat of 5.7. Cough severity was lower in Group T (*P* < 0.001). MAP and HR changes did not differ significantly, and immediate adverse events were uncommon.

**Conclusion:**

Tegileridine 0.5 mg pretreatment reduced the incidence and severity of FIC without evidence of immediate hemodynamic instability. Further active-comparator, dose-finding, and broader safety studies are required before routine clinical adoption.

**Clinical trial registration:**

https://www.chictr.org.cn/searchproj.html, identifier: ChiCTR2500115512.

## Introduction

Intravenous bolus administration of fentanyl and its analogs is widely used during induction of general anesthesia because of their rapid onset and potent analgesic properties (1). However, a cough reflex may occur immediately after injection, with reported incidence ranging from 18% to 65% (2, 3). Although generally benign, FIC can elevate intracranial, intraocular, and intra-abdominal pressures and even lead to upper airway obstruction, making it clinically desirable to prevent this adverse reaction (4–7).

Multiple pharmacological and non-pharmacological strategies have been investigated to mitigate FIC (8). Agents such as lidocaine, N-methyl-D-aspartate receptor antagonists, propofol, α2-agonists, β2-agonists, fentanyl priming, and slow injection can reduce the incidence of FIC, yet their efficacy and safety remain controversial and none have been universally adopted in clinical practice (9, 10).

Tegileridine (SHR8554) is a novel μ-opioid receptor-biased agonist that selectively activates the G-protein pathway and exhibits weak β-arrestin-2 signaling (11). It was developed to preserve analgesic efficacy while potentially reducing some opioid-related adverse effects (12). A phase 3 clinical trial in postoperative pain showed effective analgesia and an acceptable safety profile (13). Given its pharmacological profile, we hypothesized that tegileridine pretreatment might suppress FIC. To our knowledge, no randomized study has evaluated this question.

Therefore, we conducted a randomized, double-blind, placebo-controlled clinical trial to assess whether intravenous tegileridine pretreatment reduces the incidence and severity of fentanyl-induced cough during anesthetic induction.

## Methods

### Study design and ethical approval

This prospective, single-center, randomized, double-blind, placebo-controlled clinical trial was conducted at the Department of Anesthesiology of Weifang People's Hospital. The protocol was approved by the Medical Scientific Research Ethics Committee of Weifang People's Hospital (approval No. KYLL20251219-4) and prospectively registered with the Chinese Clinical Trial Registry under identifier ChiCTR2500115512 on December 26, 2025, before the enrollment of the first participant in January 2026. The study was designed and reported in accordance with the Consolidated Standards of Reporting Trials (CONSORT) guidelines and adhered to the principles of the Declaration of Helsinki. Written informed consent was obtained from all participants.

### Participants

From January 2026 to March 2026, adult patients aged 18–65 years with American Society of Anesthesiologists physical status I–III and a body mass index (BMI) between 18 and 28 kg/m^2^ who were scheduled for elective gynecological, gastrointestinal, urological, or orthopedic surgery under general anesthesia were eligible. Exclusion criteria included respiratory tract infection, chronic cough, asthma, smoking within 1 year, use of angiotensin-converting enzyme inhibitors, opioid or antitussive medications, known allergy to fentanyl or tegileridine, and pregnancy.

### Randomization and interventions

An independent research coordinator who was not involved in recruitment, clinical care, or outcome assessment generated and maintained the computer-generated randomization sequence. Participants were allocated in a 1:1 ratio to receive tegileridine 0.5 mg (Group T) or an equal volume of normal saline (Group C). Allocation concealment was implemented using sequentially numbered, opaque, sealed envelopes. After enrollment, the corresponding envelope was opened and the assignment was disclosed only to a pharmacist not involved in patient care, who prepared the allocated solution to a final volume of 5 mL in identical syringes labeled only with the participant code. Participants, anesthesiologists, outcome observers, and data analysts remained blinded to group assignment until completion of the analysis.

Each study solution was administered as a slow intravenous injection over 10 s. Immediately after completion of the injection, fentanyl 4 μg·kg^−1^ was injected rapidly as a bolus over 2 s. A blinded observer recorded cough episodes within 2 min after fentanyl injection. The 0.5-mg dose and the 10-s injection schedule immediately before fentanyl administration replicated our unpublished institutional pilot protocol. The selected dose was deliberately conservative: it was intended as a low experimental pretreatment dose during induction, lower than the 0.75- and 1.0-mg loading doses evaluated for postoperative analgesia (11, 13), and compatible with routine induction workflow. This dose should not be interpreted as the optimal prophylactic dose. Because no pharmacodynamic or dose-response data are available for tegileridine in the prevention of FIC, the minimum effective dose, ceiling effect, and optimal pretreatment interval remain unknown and require dedicated dose-finding studies. Normal saline was selected as the placebo because no single preventive intervention has been universally adopted as standard care for FIC. The placebo-controlled design enabled an initial proof-of-concept evaluation under a standardized rapid-bolus fentanyl protocol, but it was not designed to determine whether tegileridine is superior or non-inferior to established prophylactic strategies.

All patients fasted for at least 8 h and received no premedication before surgery. Standard monitoring (electrocardiography, non-invasive blood pressure, pulse oximetry, and bispectral index [BIS]) was instituted on arrival in the operating room, and patients were preoxygenated with 100% oxygen for 3 min. After completion of the 2-min observation period, anesthesia was induced with propofol 2 mg·kg^−1^ and rocuronium 0.6 mg·kg^−1^. Anesthesia was maintained with sevoflurane (end-tidal concentration 1–2%) in an oxygen/air mixture (50:50) and a remifentanil infusion titrated to maintain MAP and HR within 20% of baseline values and a BIS between 40 and 60. Mechanical ventilation was adjusted to keep end-tidal carbon dioxide between 35 and 45 mmHg. At the conclusion of surgery, sevoflurane and remifentanil were discontinued. Neuromuscular blockade was reversed using sugammadex injection, and patients were extubated when fully awake.

### Outcome measures

The primary endpoint was the incidence of FIC, defined as the proportion of patients with any cough during the 2-min observation period after fentanyl injection. Secondary endpoints included cough severity (mild, 1–2 coughs; moderate, 3–5 coughs; severe, ≥6 coughs) (14), changes in mean arterial pressure (MAP) and heart rate (HR) assessed at baseline immediately before study drug injection (T0) and at 2 min after fentanyl injection (T1), and immediate adverse events. During the 2-min observation period after fentanyl injection, adverse events of interest were recorded, including truncal rigidity, hypoxemia, apnea, nausea, and vomiting. Postoperative nausea and vomiting, depth of sedation after induction, respiratory depression beyond this observation window, and recovery characteristics were not prespecified outcome measures and were not systematically collected.

### Sample size calculation

The sample size was calculated using PASS software Version 2021 on the basis of unpublished institutional pilot data obtained before the main trial. In the pilot study, FIC occurred in 9 of 30 participants (30.0%) in the saline group and 4 of 31 participants (12.9%) in the tegileridine group. With a two-sided α level of 0.05, 80% power, and a 1:1 allocation ratio, a minimum of 88 evaluable participants was required per group. After allowing for an anticipated dropout margin of approximately 20%, the planned sample size was set at 105 participants per group. A total of 232 patients were screened, of whom 216 were randomized and analyzed (108 per group).

### Statistical analysis

Statistical analyses were performed using R software, version 4.4.3. Continuous variables are presented as mean ± standard deviation and were compared using independent-samples *t*-tests. Categorical variables are presented as number (%) and were compared using χ^2^ tests or Fisher's exact tests, as appropriate. The primary endpoint was analyzed using a χ^2^ test, with relative risk (RR), absolute risk reduction (ARR), number needed to treat (NNT), and corresponding 95% confidence intervals calculated. Cough severity was analyzed using the Cochran-Armitage trend test across ordered severity categories. Changes in MAP and HR were assessed using linear mixed-effects models with group, time, and group × time interaction as fixed effects and participant as a random intercept. Secondary endpoints were exploratory, and no adjustment for multiple comparisons was applied. A two-sided *P* < 0.05 was considered statistically significant.

## Results

### Participant flow

**Figure 1:**
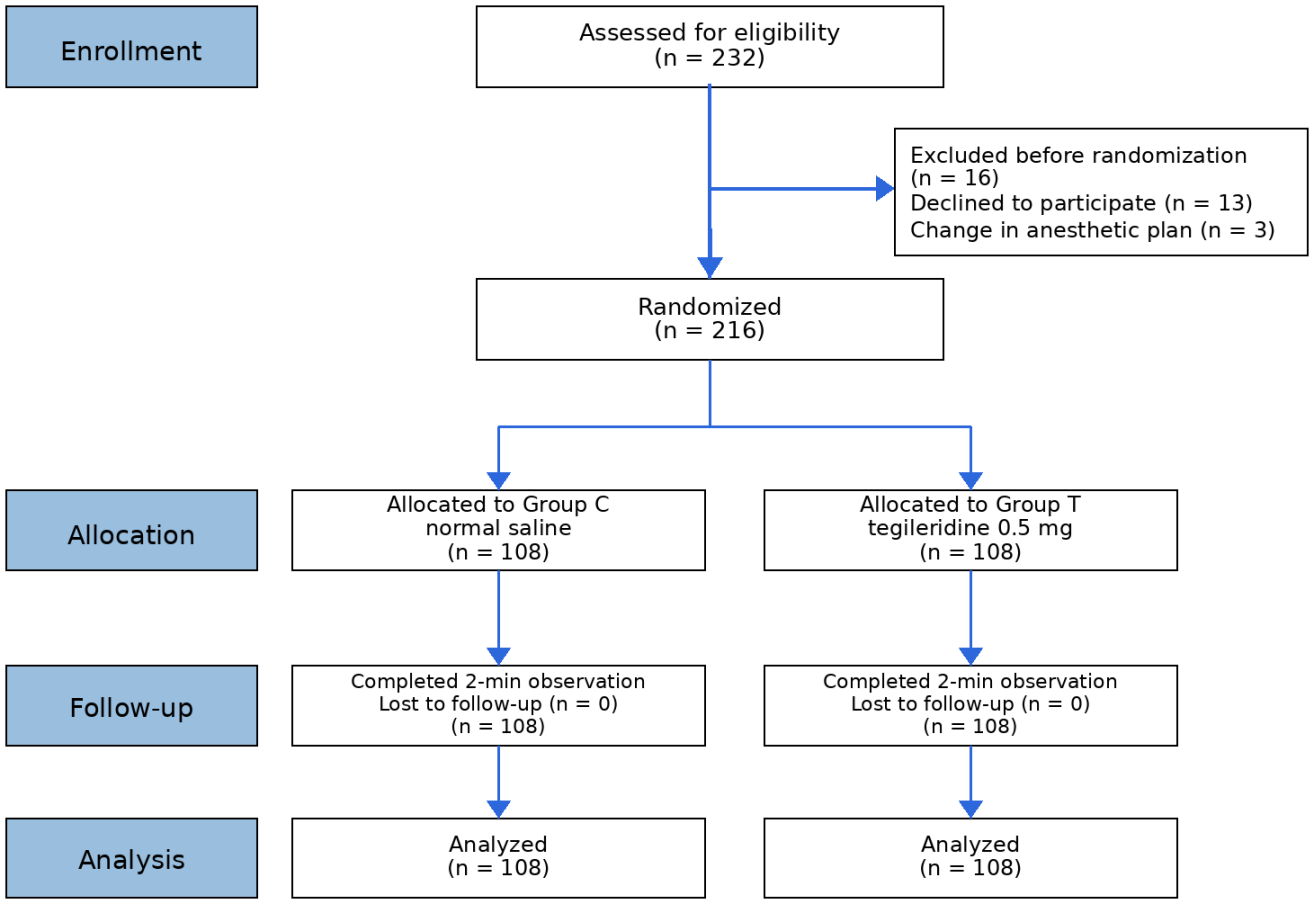
Study flowchart.

Of 232 patients assessed for eligibility, 16 were excluded before randomization: 13 declined participation and 3 had a change in anesthetic plan. The remaining 216 patients were randomized (108 to Group C and 108 to Group T), received the assigned intervention, completed follow-up through the 2-min observation period, and were included in the final analysis; no post-randomization loss to follow-up occurred (Figure 1).

### Baseline characteristics

Baseline characteristics were balanced between groups (Table 1).

**Table 1:** Baseline characteristics of patients.

Variables	Group C (*n* = 108)	Group T (*n* = 108)	*P*–value
Sex (male/female)	38/70	40/68	0.780
Age (years)	53.4 ± 12.4	52.3 ± 12.5	0.527
BMI (kg/m^2^)	23.22 ± 0.79	23.33 ± 0.95	0.336
ASA class, *n* (%)			0.740
Class I	12 (11.1)	10 (9.3)	
Class II	89 (82.4)	93 (86.1)	
Class III	7 (6.5)	5 (4.6)	
Type of surgery, *n* (%)			0.749
Gynecological surgery	17 (15.7)	14 (13.0)	
Gastrointestinal surgery	28 (25.9)	33 (30.6)	
Urological surgery	43 (39.8)	38 (35.2)	
Orthopedic surgery	20 (18.5)	23 (21.3)	

BMI, body mass index; ASA, American Society of Anesthesiologists.

### Incidence of fentanyl-induced cough

The incidence of cough differed significantly between groups (Table 2). FIC occurred in 28.7% (31/108) of patients in Group C and 11.1% (12/108) in Group T. The between-group difference was statistically significant (χ^2^ = 10.48, *P* = 0.001). Compared with the control group, tegileridine reduced the relative risk of cough by 61.3%, with an RR of 0.39 (95% CI, 0.21–0.71), an ARR of 17.6% (95% CI, 0.07–0.28), and an NNT of 5.7 (95% CI, 3.6–13.9).

**Table 2:** Incidence of fentanyl-induced cough and effect measures.

Variable	Group C (*n* = 108)	Group T (*n* = 108)	χ^2^	*P*–value	ARR (95% CI)	NNT (95% CI)	RR (95% CI)
Incidence of cough; *n* (%)	31 (28.7)	12 (11.1)	10.48	0.001	0.176 (0.07, 0.28)	5.7 (3.6, 13.9)	0.39 (0.21, 0.71)

ARR, absolute risk reduction; NNT, number needed to treat; RR, relative risk; CI, confidence interval.

### Cough severity

Cough severity also differed significantly between groups (Table 3). A Cochran-Armitage trend test across four ordered categories (no cough, mild, moderate, and severe cough) showed a significant trend toward lower cough severity in the tegileridine group (Z = 4.11, *P* < 0.001).

**Table 3:** Distribution of cough severity between groups.

Variables	Group C (*n* = 108)	Group T (*n* = 108)	Z^*^	*P*–value
Severity of cough, *n* (%)			4.11	< 0.001
No cough	77 (71.3)	96 (88.9)		
Mild	8 (7.4)	10 (9.3)		
Moderate	19 (17.6)	2 (1.9)		
Severe	4 (3.7)	0		

^*^Cochran-Armitage trend test for ordered differences across four categories: no cough, mild, moderate, and severe cough.

### Hemodynamic variables

In linear mixed-effects models, both MAP and HR decreased from baseline to 2 min after fentanyl injection in both groups. However, the between-group differences in change over time were not statistically significant. For MAP, the group × time interaction was β = −1.97 mmHg (95% CI, −5.63 to 1.69; *P* = 0.291). For HR, the corresponding estimate was β = −2.81 beats/min (95% CI, −7.07 to 1.44; *P* = 0.195) (Table 4).

**Table 4:** Hemodynamic variables at baseline and 2 min after fentanyl injection.

Parameter	Time	Group C (*n* = 108)	Group T (*n* = 108)	Group × time interaction β (95% CI)	*P*–value (group × time)
MAP, mmHg	T0	90.06 ± 9.58	89.45 ± 10.58	−1.97 (−5.63 to 1.69)	0.291
	T1	81.13 ± 11.53	78.56 ± 11.64		
HR, beats/min	T0	79.28 ± 15.18	80.71 ± 11.99	−2.81 (−7.07 to 1.44)	0.195
	T1	74.37 ± 17.68	72.99 ± 17.67		

Data at T0 and T1 are presented as mean ± SD. T0, baseline immediately before study drug injection; T1, 2 min after fentanyl injection. β represents the estimated between-group difference in change (Group T – Group C) derived from linear mixed-effects models including fixed effects for group, time, and group × time interaction, with participant as a random intercept.

### Adverse events

During the 2-min observation period after fentanyl injection, one participant in Group C developed truncal rigidity and no participant in Group T did (0.9% vs. 0%; Fisher's exact test, *P* = 1.000). No hypoxemia, apnea, nausea, or vomiting was observed in either group. No serious adverse events were observed during this observation period (Table 5).

**Table 5:** Adverse events during the 2-min observation period after fentanyl injection.

Adverse event	Group C (*n* = 108)	Group T (*n* = 108)	*P* value
Truncal rigidity	1 (0.9)	0	1.000
Hypoxemia	0	0	-
Apnea	0	0	-
Nausea	0	0	-
Vomiting	0	0	-

Data are presented as n (%). Adverse events were recorded during the 2-min observation period after fentanyl injection. The *P* value for truncal rigidity was calculated using Fisher's exact test.

## Discussion

This randomized, double-blind trial found a lower incidence and severity of fentanyl-induced cough after tegileridine 0.5 mg pretreatment than after saline. The absolute risk reduction was 17.6%, corresponding to a number needed to treat of 5.7. MAP and HR trajectories did not differ significantly between groups, and immediate adverse events during the 2-min observation period after fentanyl injection were uncommon. These findings support a potential prophylactic signal, but they should be interpreted in light of the placebo-controlled design, the single tested dose, the restricted safety window, and the selected study population.

FIC is likely multifactorial (15). Proposed contributors include stimulation of rapidly adapting receptors and pulmonary vagal C-fibers (16), activation of the pulmonary chemoreflex (14), release of histamine and neuropeptides (17), and possible effects of the citrate formulation of fentanyl preparations (18). Risk is also influenced by dose, injection speed, route of administration, age, and other patient factors (15, 19). The placebo incidence in our study (28.7%) was close to the approximately 31% control incidence reported in meta-analytic data (8).

Normal saline was selected as the comparator for this first clinical evaluation because no single prophylactic method has been universally accepted as standard care for FIC. A placebo-controlled design was therefore chosen to isolate the effect of tegileridine under a standardized rapid-bolus fentanyl protocol before undertaking larger comparative trials. Established preventive strategies include lidocaine (20, 21), NMDA receptor antagonists such as ketamine (22, 23), propofol (24, 25), α2-agonists such as dexmedetomidine (26, 27), β2-agonists (28, 29), fentanyl priming (30, 31), and slow injection of fentanyl (32). These interventions have been associated with reduced opioid-induced cough in previous studies and meta-analyses (8–10), but their effectiveness, safety, and practical burden vary. For example, pharmacological pretreatments may introduce hemodynamic or sedative effects, whereas slow injection and priming strategies may prolong or complicate induction and may add opioid exposure (33). Accordingly, the present results should position tegileridine as a candidate prophylactic option requiring further comparison with established methods, not as evidence of superiority over them. Because no active comparator was included, the relative effectiveness of tegileridine vs. lidocaine, dexmedetomidine, ketamine, fentanyl priming, or slow fentanyl injection remains unknown.

Dose selection also requires cautious interpretation. The selected 0.5-mg dose and immediate 10-s pretreatment schedule were informed by our unpublished institutional pilot data and by workflow considerations. The dose is lower than the 0.75- and 1.0-mg loading doses evaluated for postoperative analgesia, while published pooled clinical-development data include limited exposure to a 0.5-mg loading dose (11, 13). We therefore considered 0.5 mg a reasonable low experimental pretreatment dose intended to limit additional opioid exposure during induction. However, the present trial was not designed to identify the optimal dose, dose-response relationship, or optimal pretreatment interval. Dedicated dose-finding studies are needed to determine whether lower or higher doses, different dilution volumes, or different pretreatment intervals provide a better balance between antitussive efficacy and safety.

The safety interpretation of this trial is limited. Available clinical-development evidence, including data from healthy volunteers and postoperative analgesia studies, suggests that tegileridine is generally well tolerated, although nausea and vomiting remain recognized opioid-related adverse effects and the dosing regimens differed from that used in the present study (11–13). Our trial assessed only immediate adverse events during the 2-min observation period after fentanyl injection. We did not systematically evaluate postoperative nausea and vomiting, depth or duration of sedation, delayed respiratory depression, recovery characteristics, or other postoperative outcomes. Therefore, the absence of safety signals in this short window should not be extrapolated to the overall perioperative or postoperative safety profile of tegileridine. Larger trials with structured intraoperative and postoperative safety follow-up are required.

The mechanistic interpretation should remain hypothesis-generating. Tegileridine's biased agonism offers a possible biological rationale for the observed clinical findings, but the present clinical trial did not directly evaluate μ-opioid receptor signaling, β-arrestin recruitment, receptor occupancy, airway sensory nerve activity, or inflammatory mediators. Accordingly, the findings neither confirm nor refute the hypothesis that biased μ-opioid receptor signaling mediates suppression of opioid-induced cough. β-arrestin signaling has not been established as a mediator of FIC (34). Together with recent data on oliceridine pretreatment for fentanyl-induced cough (35), our results raise the possibility that biased μ-opioid receptor agonists may influence opioid-related tussive responses, but this remains speculative and requires dedicated mechanistic studies.

Several nonexclusive hypotheses may account for the lower cough incidence observed after tegileridine pretreatment. Tegileridine may partially occupy μ-opioid receptors before the fentanyl bolus, potentially modifying a proposed receptor-mediated tussive trigger (15, 36). Prior opioid exposure may also alter the responsiveness of peripheral airway afferents, including rapidly adapting receptors and vagal C-fiber terminals (37). These explanations are speculative because the present trial measured clinical cough events only and did not measure receptor occupancy, neurotransmitter release, cough-reflex sensitivity, or biomarkers.

Several limitations warrant emphasis. First, external validity is limited because the trial was conducted at a single center and enrolled relatively healthy adults with ASA physical status I–III and BMI 18–28 kg/m^2^, while excluding current or recent smokers, patients with respiratory tract infection, chronic cough, asthma, chronic respiratory disease, opioid or antitussive use, and pregnancy. The results therefore cannot be readily extrapolated to patients at higher baseline risk of FIC or to broader perioperative populations, including patients with significant pulmonary disease, obesity, frailty, or emergency surgery. Second, only one tegileridine dose and one pretreatment interval were evaluated, so the minimum effective dose, dose-response relationship, ceiling effect, and optimal timing remain unknown. Third, the observation window for adverse events was limited to 2 min after fentanyl injection, and the trial was not powered to detect rare or delayed adverse events; postoperative nausea and vomiting, sedation, delayed respiratory depression, and recovery profiles were not systematically assessed. Fourth, the absence of an active comparator such as lidocaine, dexmedetomidine, ketamine, fentanyl priming, or a slow-injection strategy prevents conclusions about the relative effectiveness or practical advantages of tegileridine among available options. Finally, mechanistic biomarkers were not measured, so the proposed biological explanations remain untested hypotheses.

Overall, these findings support further evaluation of tegileridine as a potential prophylactic option for FIC. Future studies should include multicenter enrollment, broader and higher-risk populations, dose-finding designs, structured perioperative safety assessment, and direct comparisons with established prophylactic strategies to clarify its clinical role.

## Conclusion

Tegileridine 0.5 mg pretreatment reduced the incidence and severity of fentanyl-induced cough without evidence of additional immediate hemodynamic instability in this randomized controlled trial. These findings warrant further evaluation of tegileridine as a potential prophylactic option during anesthesia induction. Multicenter dose-finding studies, active-comparator trials, mechanistic studies, and trials with broader safety follow-up are needed before routine clinical adoption can be recommended.

## Data Availability

The raw data supporting the conclusions of this article will be made available by the authors, without undue reservation.

## References

[1] Schug SA Ting S. Fentanyl formulations in the management of pain: an update. Drugs. (2017) 77:747–63. doi: 10.1007/s40265-017-0727-z 28337672

[2] Nouri N Pournajafian A Tahzibi S Djalali Motlagh S. Prevention of fentanyl induced cough by ketorolac: a prospective, randomized, and double-blind study. Anesth Pain Med. (2025) 15:e161218. doi: 10.5812/aapm-161218 41103679 PMC12524098

[3] Lin C-S Sun W-Z Chan W-H Lin C-J Yeh H-M Mok MS. Intravenous lidocaine and ephedrine, but not propofol, suppress fentanyl-induced cough. Can J Anesth. (2004) 51:654–9. doi: 10.1007/BF03018421 15310631

[4] Tweed WA Dakin D. Explosive coughing after bolus fentanyl injection. Anesth Analg. (2001) 92:1442–3. doi: 10.1097/00000539-200106000-00018 11375822

[5] Ambesh SP Singh N Gupta D Singh PK Singh U. A huffing manoeuvre, immediately before induction of anaesthesia, prevents fentanyl-induced coughing: a prospective, randomized, and controlled study. Br J Anaesth. (2010) 104:40–3. doi: 10.1093/bja/aep333 19933512

[6] Lim KJ Lee SK Lee HM Park EY Kim MH Kim YS . Aspiration pneumonia caused by fentanyl-induced cough -a case report-. Korean J Anesthesiol. (2013) 65:251. doi: 10.4097/kjae.2013.65.3.251 24101960 PMC3790037

[7] Ambesh S Singh N Srivastava K. Fentanyl induced coughing caused life-threatening Airway Obstruction in a patient with arteriovenous malformation of tongue and hypopharynx. Internet J Anesthesiol. (2008) 20. doi: 10.5580/2

[8] Kim JE Min SK Chae YJ Lee YJ Moon BK Kim JY. Pharmacological and nonpharmacological prevention of fentanyl-induced cough: a meta-analysis. J Anesth. (2014) 28:257–66. doi: 10.1007/s00540-013-1695-4 23958914

[9] Dong Y Chang X. Comparison of five prophylactically intravenous drugs in preventing opioid-induced cough: a Bayesian network meta-analysis of randomized controlled trials. Front Pharmacol. (2021) 12:684276. doi: 10.3389/fphar.2021.684276 34867314 PMC8635493

[10] Shuying L Ping L Juan N Dong L. Different interventions in preventing opioid-induced cough: a meta-analysis. J Clin Anesth. (2016) 34:440–7. doi: 10.1016/j.jclinane.2016.05.034 27687431

[11] Dhillon S. Tegileridine: first approval. Drugs. (2024) 84:717–20. doi: 10.1007/s40265-024-02033-4 38771484

[12] Shi R Chai Y Feng H Xie L Zhang L Zhong T . Study of the mass balance, biotransformation and safety of [14C]SHR8554, a novel μ-opioid receptor injection, in healthy Chinese subjects. Front Pharmacol. (2023) 14:1231102. doi: 10.3389/fphar.2023.1231102 37781692 PMC10538116

[13] Wang T Wang Y Xie H Wu Z Yu S Ou Y . Tegileridine for moderate-to-severe acute pain following abdominal surgery: a randomized, double-blind, phase 3 clinical trial. Cell Rep Med. (2025) 6:102477. doi: 10.1016/j.xcrm.2025.102477 41365300 PMC12765830

[14] Böhrer H Fleischer F Werning P. Tussive effect of a fentanyl bolus administered through a central venous catheter. Anaesthesia. (1990) 45:18–21. doi: 10.1111/j.1365-2044.1990.tb14496.x 2316832

[15] Chen R Tang LH Sun T Zeng Z Zhang YY Ding K . Mechanism and management of fentanyl-induced cough. Front Pharmacol. (2020) 11:584177. doi: 10.3389/fphar.2020.584177 33324214 PMC7723435

[16] El Baissari MCT Taha SK Siddik-Sayyid SM. Fentanyl-induced cough–pathophysiology and prevention. Middle East J Anaesthesiol. (2014) 22:449–56. 25137861

[17] Kamei J Nakanishi Y Asato M Ikeda H. Fentanyl enhances the excitability of rapidly adapting receptors to cause cough via the enhancement of histamine release in the airways. Cough. (2013) 9:3. doi: 10.1186/1745-9974-9-3 23369146 PMC3566957

[18] Nurmi HM Lätti AM Brannan JD Koskela HO. Comparison of mannitol and citric acid cough provocation tests. Respir Med. (2019) 158:14–20. doi: 10.1016/j.rmed.2019.09.011 31542680

[19] Liu MQ Li FX Han YK He JY Shi HW Liu L . Administration of fentanyl via a slow intravenous fluid line compared with rapid bolus alleviates fentanyl-induced cough during general anesthesia induction. J Zhejiang Univ Sci B. (2017) 18:955–62. doi: 10.1631/jzus.B1600442 29119733 PMC5696314

[20] Phuvachoterojanaphokin N Watanaboonyongcharoen G Jinawong S Munjupong S. Low-dose lidocaine attenuates fentanyl-induced cough: a double-blind randomized controlled trial. Eur J Clin Pharmacol. (2022) 78:813–21. doi: 10.1007/s00228-022-03282-6 35089372

[21] Tan W Li S Liu X Gao X Huang W Guo J . Prophylactic intravenous lidocaine at different doses for fentanyl-induced cough (FIC): a meta-analysis. Sci Rep. (2018) 8:9946. doi: 10.1038/s41598-018-27457-3 29967371 PMC6028622

[22] Hung KC Yu TS Hsu CW Liu WC Wu JY Liao SW . Efficacy and safety of ketamine and esketamine for preventing opioid-induced cough: a systematic review and meta-analysis of randomized controlled trials. Syst Rev. (2025) 14:131. doi: 10.1186/s13643-025-02886-0 40598615 PMC12211305

[23] Honarmand A Safavi M Cheshmaviz N. Effect of different doses of ketamine on fentanyl-induced cough. AACC. (2024) 10. doi: 10.18502/aacc.v10is2.17209

[24] Firouzian A Emadi S Baradari A Mousavi R Kiasari A. Can low dose of propofol effectively suppress fentanyl-induced cough during induction of anaesthesia? A double blind randomized controlled trial. J Anaesthesiol Clin Pharmacol. (2015) 31:522. doi: 10.4103/0970-9185.169082 26702212 PMC4676244

[25] Sedighinejad A Naderi Nabi B Haghighi M Imantalab V Hadadi S Erfani Sayar R . Propofol is effective to depress fentanyl-induced cough during induction of anesthesia. Anesth Pain. (2013) 2:170–3. doi: 10.5812/aapm.8383 24223355 PMC3821139

[26] Zhou W Zhang D Tian S Yang Y Xing Z Ma R . Optimal dose of pretreated-dexmedetomidine in fentanyl-induced cough suppression: a prospective randomized controlled trial. BMC Anesthesiol. (2019) 19:89. doi: 10.1186/s12871-019-0765-z 31153360 PMC6545214

[27] He L Xu J-M Dai R-P. Dexmedetomidine reduces the incidence of fentanyl-induced cough: a double-blind, randomized, and placebo-controlled study. Ups J Med Sci. (2012) 117:18–21. doi: 10.3109/03009734.2011.629749 22335390 PMC3282237

[28] Agarwal A Azim A Ambesh S Bose N Dhiraj S Sahu D . Salbutamol, beclomethasone or sodium chromoglycate suppress coughing induced by iv fentanyl. Can J Anesth. (2003) 50:297–300. doi: 10.1007/BF03017801 12620955

[29] Ping-Wing L Chung-Hsi H Ya-Churn C. Terbutaline inhalation suppresses fentanyl-induced coughing. Can J Anaesth. (1996) 43:1216–9. doi: 10.1007/BF03013427 8955969

[30] Shrestha S Bhattarai B Shah R. Preemptive use of small dose fentanyl suppresses fentanyl induced cough. Kathmandu Univ Med J. (2014) 10:16–9. doi: 10.3126/kumj.v10i4.10988 23575046

[31] Du BX Cao L Zhao WL Xu ZH Song J Shi XY. Pre-emptive small dose of fentanyl suppresses fentanyl-induced cough: a meta-analysis of randomized controlled trials. Int J Clin Exp Med. (2014) 7:826–36. 24955151 PMC4057830

[32] Yu H Yang X-Y Zhang X Li Q Zhu T Wang Y . The effect of dilution and prolonged injection time on fentanyl-induced coughing. Anaesthesia. (2007) 62:919–22. doi: 10.1111/j.1365-2044.2007.05147.x 17697219

[33] Cheng XY Lun XQ Li HB Zhang ZJ. Butorphanol suppresses fentanyl-induced cough during general anesthesia induction: a randomized, double-blinded, placebo-controlled clinical trial. Medicine. (2016) 95:e3911. doi: 10.1097/MD.0000000000003911 27367987 PMC4937901

[34] Mafi A Kim S-K Goddard WA. Mechanism of β-arrestin recruitment by the μ-opioid G protein-coupled receptor. Proc Natl Acad Sci USA. (2020) 117:16346–55. doi: 10.1073/pnas.1918264117 32601232 PMC7368253

[35] Xing RY Gong WY Li CG Mamtili I Zhu SX Zhou WJ . Oliceridine effectively attenuates fentanyl-induced cough during general anesthesia induction. Front Med. (2026) 13:1786137. doi: 10.3389/fmed.2026.1786137 PMC13215988 42221070

[36] Zhang J Zhang D Liu Y Yu W Lin Y Hua F . Effects of remifentanil pretreatment on sufentanil-induced cough suppression during the induction of general anesthesia. J Perianesth Nurs. (2025) 40:90–4. doi: 10.1016/j.jopan.2024.03.015 39023477

[37] Gu C Zhou M Wu H Li F Tang Q. Effects of different priming doses of fentanyl on fentanyl-induced cough: a double-blind, randomized, controlled study. Pharmacol Rep. (2012) 64:321–5. doi: 10.1016/S1734-1140(12)70771-5 22661182

